# Towards Precision Oncology: Enhancing Cancer Screening, Diagnosis and Theragnosis Using Artificial Intelligence

**DOI:** 10.3390/curroncol29080449

**Published:** 2022-08-12

**Authors:** William T. Tran

**Affiliations:** 1 Department of Radiation Oncology, Temerty Faculty of Medicine, University of Toronto, Toronto, ON M4N3M5, Canada; william.tran@sunnybrook.ca; 2 Department of Radiation Oncology, Sunnybrook Health Sciences Centre, Toronto, ON M4N3M5, Canada; 3 Biological Sciences Platform, Sunnybrook Research Institute, Toronto, ON M4N3M5, Canada

## 1. Introduction

Highly complex and multi-dimensional medical data containing clinical, radiologic, pathologic, and sociodemographic information have the potential to advance precision oncology. There is a growing demand to identify meaningful and actionable biomarkers coupled with other patient-related data to improve detection and treatment strategies, ultimately yielding signatures that can enhance prognostic endpoints and individualize patient care. We are in an exciting time when increased computational capacity and advances in artificial intelligence (AI) are affording new frontiers in cancer research, treatment, and risk stratification.

## 2. Research and Clinical Topics Covered: Cancer Screening, Diagnosis and Theragnosis in *Current Oncology*

In this special issue of *Current Oncology*, we present a collection of research articles focused on enhancing precision oncology using artificial intelligence. The contributions cover various cancer disease sites, including breast, central nervous system, cervical, colorectal, and prostate cancers. These reports also comprise topics in computer vision and machine learning (ML), which were used in radiology and digital pathology image analysis, feature extraction, segmentation, and classification tasks. Additionally, several concepts in tailoring AI techniques to gain insight into important clinical endpoints were reviewed.

Lagree et al. [1] presented work on developing a computer-aided diagnosis (CAD) system in pathology to classify breast cancer Nottingham grade. This study implemented convolutional neural networks (CNNs) for tumor segmentation of digitized breast biopsies, followed by machine learning techniques for feature extraction within the regions of interest (ROIs) containing tumor cells. Imaging biomarkers yielded from density, graph, and count features were inputted to carry out ML classification. Ensemble models integrated both imaging and clinical features to evaluate ML model performances and predict tumor grade. The study results showed an area under the curve (AUC) of 0.836 using computationally-derived and clinical features into models. Although further validation is needed, the study results demonstrate promising ways to expedite diagnosis and reporting standard tumor characteristics using computer analysis.

Li et al. [2] explored deep-learning techniques to detect malignant cells from digitized cervical cytology samples (i.e., positive cervical cancer). The dataset comprised 800 whole slide images, constituting > 200,000 image patches retrieved from the *Digital Human Body* Vision Challenge (Alibaba Cloud TianChi Company). A proposed deformable and global context aware (DGCA) region-based convolutional neural network (RCNN) model was developed based on an RCNN feature pyramid network (FPN) architecture. The DGCA-RCNN model showed the highest classification performance among all of the studied models, yielding an optimal AUC of 0.670. The model’s strengths include model scalability, detection of cells that exhibit varying morphologies (e.g., size and clusters), and consideration of spatial context information to improve performances.

Russo et al. [3] investigated a radiomics-guided grading algorithm for central nervous system tumors. Imaging data were collected from 11[C]-MET PET/CT and pre-processed before analyses. The dataset comprised a significant proportion of glioblastomas (*n* = 33) among other histologic types (*n* = 56; all tumors). Radiomics analyses were carried out on segmented ROIs and volumes of interest (VOIs) based on operator evaluation and using the standard uptake value (SUV). There were 44 radiomics features extracted, including texture indices, histogram indices, shape descriptors, and conventional feature sets. A discriminant analysis was used for classification. A maximum accuracy of 84.98% (AUC 78.91%) was achieved in grading high- versus low-grade CNS tumors. The potential impact of this work includes AI-enabled lesion detection and classification that may also help guide treatment approaches (e.g., radiotherapy) and potentially lead to image-guided response monitoring systems in oncology.

Cosmin Secasan et al. [4] showed the results of their radiomics study on prostate cancer. Their experiments consisted of an AI system pipeline to predict malignant lesions from ultrasound (US) shear-wave elastography. A prospective dataset of US imaging data from 356 patients was assembled, of which ~60% of patients were confirmed for prostate cancer using standard pathology as ground truth labels. Prostate scans were partitioned into several target zones, and tissue stiffness (i.e., rigidity) was measured within the zones using a *mean elasticity* parameter. Multiple machine learning models, including logistic regression (LR), decision tree classifier, and dense neural network (DNNs), were trialed. Their ensemble model yielded a prediction accuracy of 98%. This work demonstrates the importance of future radiomics approaches to enhance non-invasive diagnostic techniques.

Qiu et al. [5] presented a review of potential applications of AI in colorectal cancer (CRC) screening, diagnosis and theragnosis. AI-driven screening opportunities include high accuracy stratification of high-risk CRCs, polyp classification, and lesion localization. In CRC diagnosis, AI frameworks from several studies were capable of classifying tumor subtypes and predicting distant hepatic metastases based on magnetic resonance imaging (AUC = 0.94). Moreover, theragnostic tools were discussed, highlighting machine learning algorithms to predict pathologic complete response (PCR) to neoadjuvant radiation and chemotherapy. Previous studies have also shown artificial neural networks (ANNs), k-nearest neighbor (k-NN), support vector machines (SVMs), and random forest (RF) models (among others), which demonstrated high performances in predicting response to therapy. Lastly, prognostic models were framed as an important area of AI research in CRC. Recent research has included deep-learning analysis such as, convolutional neural networks (CNNs) on pathology samples, and have yielded promising signals associated with survival outcomes. The authors also recapitulate the need to refine staging information, which could be achieved with AI to gain better insight for risk stratification, and for guiding optimal treatments and prognostication.

## 3. Discussion and Outlook

These studies represent the ongoing research synergy between scientists and clinicians. They also demonstrate the expansion of opportunities in AI-focused precision oncology. However, several considerations will need to be carried for successful clinical translation and implementation. These include a system built on careful curation of input data (i.e., standardization and unbiased data representation of real-world populations), developing reproducible and repeatable modelling (e.g., transparency in hyperparameters), and extensive validation. Artificial intelligence will also have to move at the same pace as contemporary developments in cancer treatments, diagnostic procedures and imaging technologies. These factors can affect prognosis, tumor biology and thus, the structure and dimensionality of input data used for AI analysis.

Many studies are still undergoing proof-of-concept testing [6]. To address the need for good practices in AI reporting in clinical research, the EQUATOR Network is currently updating the TRIPOD statement and the PROBAST guideline tailored for AI (i.e., TRIPOD-AI and PROBAST-AI) [7]. The checklists will comprise many of the existing items used for regression-based prediction models but are poised to include specific recommendations to ML, such as guidelines for reporting supervised and unsupervised learning architecture, handling loss functions, and treating missing data within the ML pipeline [8]. TRIPOD-AI and PROBAST-AI guidelines will help build confidence in clinical AI as it is implemented into routine practice. Ultimately, governance, ethical considerations and policy development will need to be carried out to gain widespread acceptance. Existing AI systems for medicine have already been approved by the United States Food and Drug Administration (US-FDA), demonstrating great promise for AI-integrated oncology [9].

Automating medical diagnosis, image analysis, and computationally-derived biomarkers will impact the clinical workflow. In radiation oncology, automatic tumor segmentation algorithms will alleviate time-consuming tasks, such as target delineation (i.e., tumor contouring), optimizing dosimetric plans and quality control. Ultimately, this will decrease wait times for radiation treatment [6]. Artificial intelligence in radiation, medical and surgical oncology will grow with data aggregates. Careful consideration of disease-specific data complexities will also be needed. Specifically, the validation and application of models will need to be tested across various cancers.

Overall, the use of multidimensional data and multiomics analyses will impact clinical decisions and workflow by directing patients into individualized pathways according to risk stratification and prediction modelling during all care phases [9,10]. Beyond standard clinical care, developments in AI models will facilitate drug development and clinical trials. For example, strengthening screening of positive cases, randomization, identifying appropriate populations for inclusion in studies, and accounting for important factors such as race, sex, and gender for a holistic approach to patient care [10].

In conclusion, there is great promise in AI-driven oncology. Current studies focus on several disease sites, diagnostic and therapeutic approaches, and some applications are already achieving regulatory approval. With ongoing advances in medical equipment, imaging, technology and biological insight, AI will undoubtedly play a critical role in the oncologic management of patients.
